# A Link between Atmospheric Pressure and Fertility of *Drosophila* Laboratory Strains

**DOI:** 10.3390/insects12100947

**Published:** 2021-10-18

**Authors:** Natalya V. Adonyeva, Petr N. Menshanov, Nataly Gruntenko

**Affiliations:** 1 Institute of Cytology and Genetics, Siberian Branch of the Russian Academy of Sciences (SB RAS), 630090 Novosibirsk, Russia; nadon@bionet.nsc.ru (N.V.A.); eternity@bionet.nsc.ru (P.N.M.); 2 Laser Systems Department, Novosibirsk State Technical University, 630090 Novosibirsk, Russia; 3 Physiology Department, Novosibirsk State University, 630090 Novosibirsk, Russia

**Keywords:** *Drosophila*, fertility, atmospheric pressure, stress

## Abstract

**Simple Summary:**

The researchers usually keep insects under study under thoroughly controlled conditions. However, sometimes they encounter a situation where the data they obtained under such conditions demonstrate an obvious side effect of some unaccounted factor. Here we provide evidence that changes in atmospheric pressure could be responsible for some such cases.

**Abstract:**

Standardization of conditions under which insects are kept is of great importance when studying their physiology and researchers do their best to maintain it. Nevertheless, sometimes an obvious side effect of some unaccounted factor affecting insects’ reproduction can be revealed even under thoroughly controlled laboratory conditions. We faced such a phenomenon when studying the fertility level in two wild type *Drosophila melanogaster* strains. For fertility analysis, 50 newly emerged females and 50 males of each strain under study were transferred to fresh medium daily within 10 days. We found out that fertility of both strains was stable on days 2–10 after the oviposition onset in one experiment, while in another one it was significantly decreased during days 5–10. When compared to publicly available meteorological data, these changes in the fertility level demonstrated a strong association with one weather factor: barometric pressure. Thus, we conclude that changes in atmospheric pressure can be considered a factor affecting insects reproduction and discuss a possible mechanism of their influence on fertility.

## 1. Introduction

Human activity might lead to drastic climate changes. For example, an increase in global average temperature has been registered in 2018 as compared to the pre-industrial levels [1]. In nature, other basic meteorological parameters including barometric pressure are tightly linked with temperature and might be affected by human-induced climate changes as well. Gillett et al. (2003) described increases in sea-level pressure over the southern Europe and North Africa and decreases in the polar regions in response to human influence [2]; Schaller et al. found that human influence increased the risk of low pressure northwest of Britain [3]. These human-induced effects on barometric pressure raise serious concerns about the possible consequences of such barometric pressure changes on living beings.

On the other hand, the negative influence of changes of atmospheric pressure on human health has been established. Significant correlations were discovered between daily barometric pressure variation and daily stroke hospitalization [4], between low barometric pressure and aggressive behavior in patients in psychiatric hospitals [5], between low barometric pressure and increased pain and stress levels in the patients with fibromyalgia [6]. Thus, changes in atmospheric pressure as an aspect of global warming could have a serious effect on public health.

In insects, an atmospheric pressure could also affect behavior and physiology. In particular, it was shown that barometric pressure has a prominent effect on the olfactory system in *Conotrachelus nenuphar* (Coleoptera) [7]. Rapid barometric changes significantly reduced the flight initiation of female *Trichogramma evanescens* and *Trichogramma pretiosum* (Hymenoptera) [8]. In response to decreasing barometric pressure, female *Pseudaletia unipuncta* (Lepidoptera) and *Macrosiphum euphorbiae* (Hemiptera) exhibited significantly reduced calling behavior, and male *Diabrotica speciosa* (Coleoptera) exhibited decreased locomotory activity [9]. Data on reduced locomotory and mating activities under decreasing barometric pressure were obtained in other insect species as well: *Aphidius nigripes* and *Cotesia glomerata* (Hymenoptera), *Frankliniella schultzei* (Thysanoptera) and *Diaphorina citri* (Hemiptera) [10,11,12,13]. These results reveal a quick social observational learning and highlight the potential importance of meteorological conditions for mate copying, a trait potentially driving reproductive isolation. Pellegrino et al. (2013) suppose that the changes in insect behavior that occurred under decreasing barometric pressure could reduce the probability of negative effects (injury or even death) of adverse weather [9].

As for *Drosophila melanogaster*, there is evidence that changes in atmospheric pressure could affect mating behavior [14,15,16], lifespan [17,18], walking speed and flight performance [19]. Having been involved in neurohormonal stress response investigations in *D. melanogaster* model for many years [20,21], we always do our best to standardize the conditions of our research as much as possible in order to have a control group, which is not influenced by any stressors. Changes in diet or temperature of development can alter longevity and fertility as well as metabolism and hormonal levels [22,23,24]. Thus, flies in our experiments are always kept in incubators under controlled laboratory conditions. However, sometimes we face a situation where the data on a given control group demonstrate an obvious side effect of some unaccounted factor.

In this study, we investigated fertility in wild type *D. melanogaster* strains and found that a fertility drop, which occurred in one of our experiments, correlates with a decrease in atmospheric pressure. Thus, we supposed that fertility is another physiological trait in insects that could be affected by changes in atmospheric pressure including those caused by ongoing climate change.

## 2. Materials and Methods

### 2.1. Drosophila Melanogaster Strains and Rearing

Two *D. melanogaster* isofemale strains, Bi90 and 153, established from wild-caught females of Kyrgyzstan natural population in 2004 and Uzbekistan natural population in 1989, respectively, were used in this study. Both strains were interbred for more than 300 generations, so they could be considered nearly isogenic. Then, 20 generations prior to the experiments described here, one isolated pair of flies was taken from each strain and newly established Bi90 and 153 branches were treated with tetracycline for three generations to make strains *Wolbachia*-free. Flies were maintained on standard medium (agar-agar, 7 g/L; corn grits, 50 g/L; dry yeast, 18 g/L; sugar, 40 g/L) at 25 °C with 65% relative humidity and 12:12 h light:dark cycle. Adults were synchronized at eclosion (flies were collected every 3–4 h).

### 2.2. Fertility Analysis

For fertility analysis, five newly emerged females and five males were placed in vials with 7 mL of standard medium and were transferred to vials with fresh medium daily within 10 days. The sample size was 10 vials (50 females) for each strain under study. The vials were kept in an incubator until progeny appeared. The number of flies emerging from each vial was counted using the SeedCounter mobile application [25] and fertility was expressed as the number of progeny per female per day. The experiments were performed twice, in the end of March and in the beginning of July 2020 in Novosibirsk, Russia, 35 km from Tolmachevo airport.

### 2.3. Meteorological Data Analysis

The publicly available meteorological data for the Tolmachevo airport station in Ob, Novosibirsk, Russia, were obtained from the website https://www.wunderground.com (accessed on 10 September 2021). The data on these weather parameters were averaged for 24 h between the time of the transfer of flies from vial to vial.

### 2.4. Statistical Analysis

Data on fecundity were analyzed via 2-way mixed-design ANOVA (with day after eclosion as the within-subjects factor and strain as the between-subjects factor). Data on basic meteorological measurements were analyzed via 2-way nested ANOVA (with day after eclosion as the within-subjects factor nested in the between-subjects factor of experiment). The comparison of the group means in ANOVA was performed with the Benjamini–Hochberg post-hoc test. All data are presented as means ± s.e.m. Variable associations were analyzed by principal component analysis with extraction of PCs associated with variables analyzed. Pairwise associations were estimated by the Spearman rank correlation test. The results were considered significant at *p* < 0.05.

## 3. Results and Discussion

**Figure 1:**
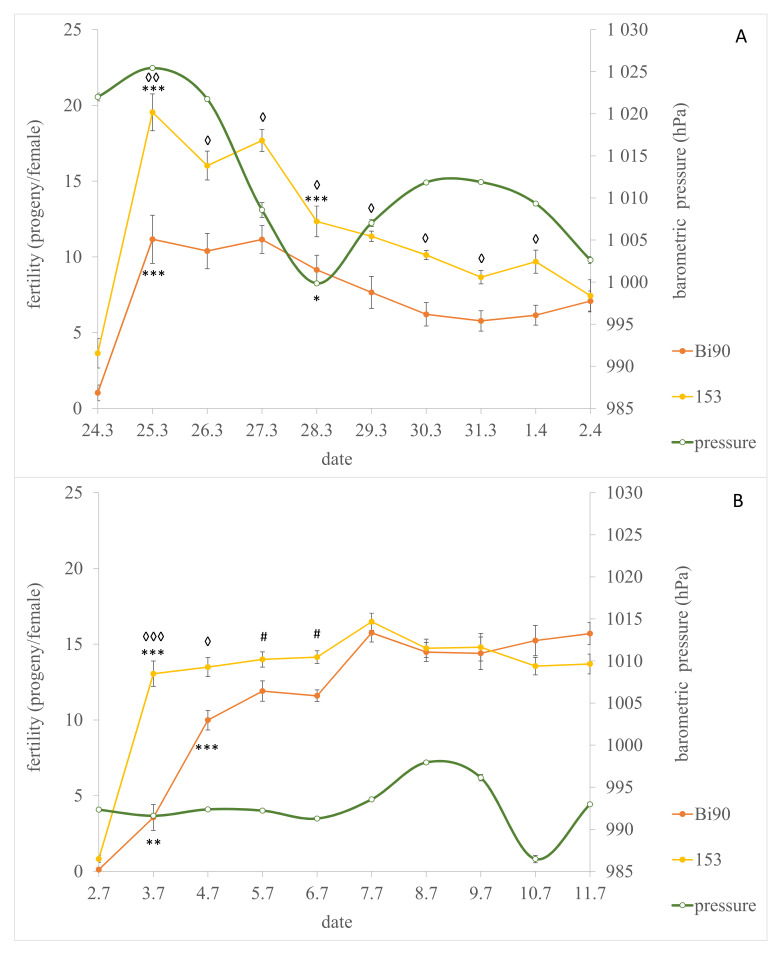
The fertility of *D. melanogaster* wild type strains Bi90 and 153 in comparison with variations of barometric pressure in Novosibirsk, Russia, in the end of March (**A**) and beginning of July (**B**) of 2020 (https://www.wunderground.com/history, (accessed on 10 September 2021)). Each point represents an average value of 10 tests (N = 5 for each test) as means ± s.e.m. The diamonds and hashtags indicate post-hoc differences between fertility levels of Bi90 and 153 flies on the same day; the asterisks illustrate the post-hoc differences in comparison with the fertility level of the same line on previous day. One diamond or asterisk indicates *p* < 0.05; two diamonds or asterisks, *p* < 0.01; three—*p* < 0.001, one hashtag indicates tendency to differences—*p* < 0.1.

Bi90 flies demonstrated lower levels of fertility in comparison with 153 flies in both experiments: during days 1–9 after eclosion (24 March—1 April) in the first experiment (Figure 1A; STRAIN × DAY-F_(9,162)_ = 4.14, *p* = 0.000082; DAY-F_(9,162)_ = 68.43, *p* < 1.0 × 10^−15^; STRAIN-F_(1,18)_ = 30.02, *p* = 0.000034) and during days 2–5 (3–6 July) in the second experiment (Figure 1B; STRAIN × DAY-F_(9,153)_ = 18.04, *p* < 1.0 × 10^−15^; DAY-F_(9,153)_ = 144.8, *p* < 1.0 × 10^−15^; STRAIN-F_(1,17)_ = 8.38, *p* = 0.011).

It should be noted that the fertility levels of both strains under the July study increased in the first days after the start of oviposition and then remained high up until the end of observations (Figure 1B). Similar to the July study, the fertility levels of our strains in the March study increased during the first day and remained high on the days 2–4. However, in contrast to July study, the fertility levels of both Bi90 and 153 went down on the day 5 of the experiment (28 March 2020) and remained low until the end of the experiment, evidencing the effect of some unaccounted factor (Figure 1A).

We tried to detect this factor and find an explanation for the difference in fertility of strains under study in these two experiments. Starting from the end of March, our region usually undergoes a period of seasonal change from winter to spring, and atmospheric pressure was already shown to affect *Drosophila* physiological phenotypes [5]. For these reasons, we analyzed the variations of basic meteorological measurements during both experiments as possible external factors. Within the studied periods, temperature (F_(1,1014)_ = 10,921, *p* < 1.0 × 10^−15^; Figure S1A), humidity (F_(1,1014)_ = 219.4, *p* < 1.0 × 10^−15^; Figure S1B), wind speed (F_(1,1014)_ = 552.2, *p* < 1.0 × 10^−15^; Figure S1C) or barometric pressure (F_(1,1014)_ = 18,651, *p* < 1.0 × 10–15; Figure S1D) were different during experiments 1 and 2, with specific patterns of changes identified. However, only barometric pressure demonstrated a specific pattern of within-day changes in the first experiment in comparison with the second one (F_(20,1014)_ = 5.88, *p* < 7.7 × 10^−15^; Figure S1E). It should be noted that a continuous rapid fall of barometric pressure was identified on 27 March 2020, which corresponds to day 4 of the first experiment (Figure S1D,E).

**Figure 2:**
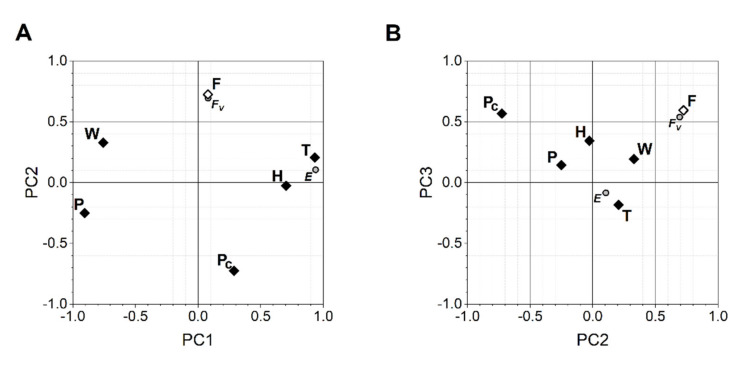
Projection of the temperature (T), humidity (H), wind speed (W), air pressure (P), air pressure change (Pc) and fertility (F) variables on (**A**) PC1 vs. PC2 and (**B**) PC2 vs. PC3 planes. The meteorological variables are marked as open diamonds, the fertility variable—as dark diamonds. Within-day fertility variance (Fv) and experiment (E) supplementary variables are marked as grey circles. Supplementary variables were not used in PCA to determine PCs. Each PC axis represents the level of association between PCs and the variables analyzed.

Principal component analysis revealed 3 PCs that determine more than 80% of all variability in meteorological data and fecundity levels (Table S1). All absolute measures of meteorological data were highly interdependent and determined by PC1, but are less connected to the levels of fertility demonstrated by flies (Figure 2). In contrast, the air pressure dynamic and within-day fertility were strongly associated with PC2 and PC3, each of which is less connected to other meteorological data included in principal component analysis (Table S1; Figure 2).

To clarify identified PCs, we included two supplementary variables into PCA (Table S1). The supplementary variable “Experiment” was strongly associated with the PC1 only and supported our ANOVA findings on the differences of temperature, humidity, wind speed and barometric pressure conditions between experiments conducted. In contrast, the supplementary variable “Within-day fertility variance” had the strongest negative as-sociation with the PC2. Since the PC2 is associated positively with the levels of fertility and negatively with the within-day levels of air pressure dynamic, the increased within-day air pressure dynamic might be a critical factor that disturbed the reproduction function in our flies. This suggestion is supported by the negative association between the fertility levels and the air pressure change levels (R = −0.38, *p* < 2.9 × 10^−8^), which was not masked by other sources of variance linked to PC1 and PC3-PC6.

To verify our conclusions, we have analyzed one more experiment, which had been performed on 17 July 2020–26 July 2020, in parallel with a case of rapid fall of barometric pressure (Figure S2). Adding these new data to our PCA model has had minimal effect on PC loadings identified previously and support our initial findings (Table S2).

The decreased mating frequency in response to rapid changes in atmospheric pressure could probably explain the significant reduction in fertility we observed in our first experiment. Moreover, the effect of atmospheric pressure on fertility was discovered earlier in *Conotrachelus nenuphar* (Coleoptera) [7]: periods of low barometric pressure increased oviposition. However, in *D. melanogaster*, mating usually occurs on the first day after eclosion and females obtain a large number of sperms, which are stored in spermathecae for up to two weeks, and become unreceptive to courting males for several days [26]. Thus, the changes in female fertility should not directly correlate with male mating activity. On the other hand, the pattern of fertility reduction in our first experiment corresponds well with the dynamics of fertility we have observed earlier in the experiments on heat stress shock influence on fertility [27]. The short-term (2 h) heat stress causes the arrest of oviposition on the day of heat exposure and the prolonged fertility decrease during the next several days as a result of a delay in oocyte development due to stress-induced hormonal imbalance [20,27]. It is not implausible that the decrease in the fertility of our flies in the first experiment on day 4 was somehow associated with changes in absolute oxygen levels induced by the continuous fall of atmospheric pressure on 27 March 2020. In humans, oxygen saturation is shown to be decreased when atmospheric pressure is decreased [28], and flies have similar O_2_ response pathways to those of mammals [29]. Bosco et al. [18], who found that *D. melanogaster* imago’s lifespan was strongly affected by hypoxia and hyperbaria, supposed that an induction of oxidative stress might explain this phenomenon. This suggestion is supported by recent human studies on hypobaric hypoxia effect on generation of reactive oxygen species [30,31]. Taking this into account, we think it possible that the drastic decrease in atmospheric pressure that occurred during our March experiment caused hypoxia and oxidative stress resulting in the fertility drop similar to that caused by heat stress.

We assume that the phenomenon we described here can be, to a certain extent, considered as a model predicting one of the possible consequences of global climate change: the latter results in a sudden reduction of barometric pressure and a consequent increase in oxidative stress levels in living beings including humans.

In light of the already identified global greenhouse effect produced by industry, our data on the altered flies’ fertility after sudden air pressure drops highlight another negative perspective of humanity-induced climate change that might impact functioning of numerous ecological chains dependent on insects worldwide.

## 4. Conclusions

We found changes in data on *D. melanogaster* fertility obtained under controlled temperature, humidity, food supply and light:dark cycle to correlate with drastic decrease in atmospheric pressure and hypothesized that oxidative stress might be a mechanism that underlies this effect.

## Data Availability

The raw data on fertility are available on request from the corresponding author. The meteorological data are available on the website https://www.wunderground.com (accessed on 10 September 2021).

## Supplementary Materials

The following are available online at https://www.mdpi.com/article/10.3390/insects12100947/s1, Figure S1: The within-day levels of (**A**) temperature, (**B**) wind speed, (**C**) humidity, (**D**) barometric pressure and (**E**) measure-to-measure atmospheric pressure changes. Figure S2: The fertility of *D. melanogaster* wild type strain Bi90 in comparison with variations of barometric pressure in Novosibirsk, Russia, in the end of July of 2020. Table S1. Principal component analysis (PCA) on meteorological data and fertility levels of Bi90 and 153 *D. melanogaster* strains. Table S2. Principal component analysis (PCA) on meteorological data and fertility level of Bi90 *D. melanogaster* strain.
