# Supplementary material for: A Link between Atmospheric Pressure and Fertility of Drosophila Laboratory Strains
Source: Insects. 2021 Oct 18;12(10):947. doi: 10.3390/insects12100947 (PMC8538592; doi:10.3390/insects12100947)
Supplement: Supplementary file 1 [file insects-12-00947-s001.zip › Figure S1.pdf]

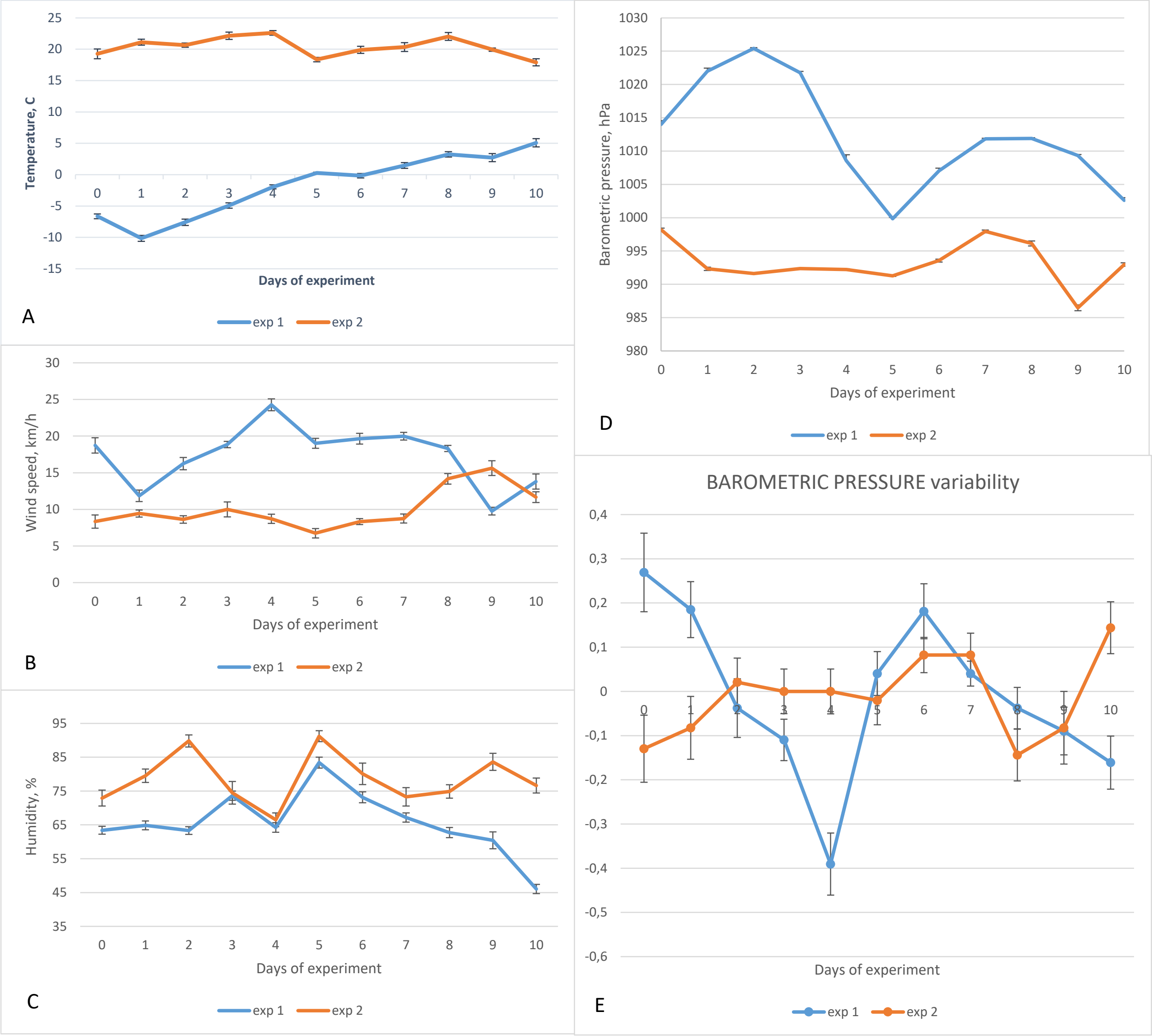

Figure S1. The within-day levels of (A) temperature, (B) wind speed, (C) humidity, (D) barometric pressure and (E) measure-to-measure atmospheric pressure changes during the first (blue, 23-03-2020 to 02-04-2020, days 0-10) and second (red, 01-07-2020 to 11-07-2020, days 0-10) experiments. Please note that the meteorological data were not counted from midnight to midnight as is shown on the website (https://www.wunderground.com/history) but from one daily transfer of flies into a new vial to the next. The data are presented as means + SEM.
