# Supplementary material for: A Link between Atmospheric Pressure and Fertility of Drosophila Laboratory Strains
Source: Insects. 2021 Oct 18;12(10):947. doi: 10.3390/insects12100947 (PMC8538592; doi:10.3390/insects12100947)
Supplement: Supplementary file 1 [file insects-12-00947-s001.zip › Figure S2.pdf]

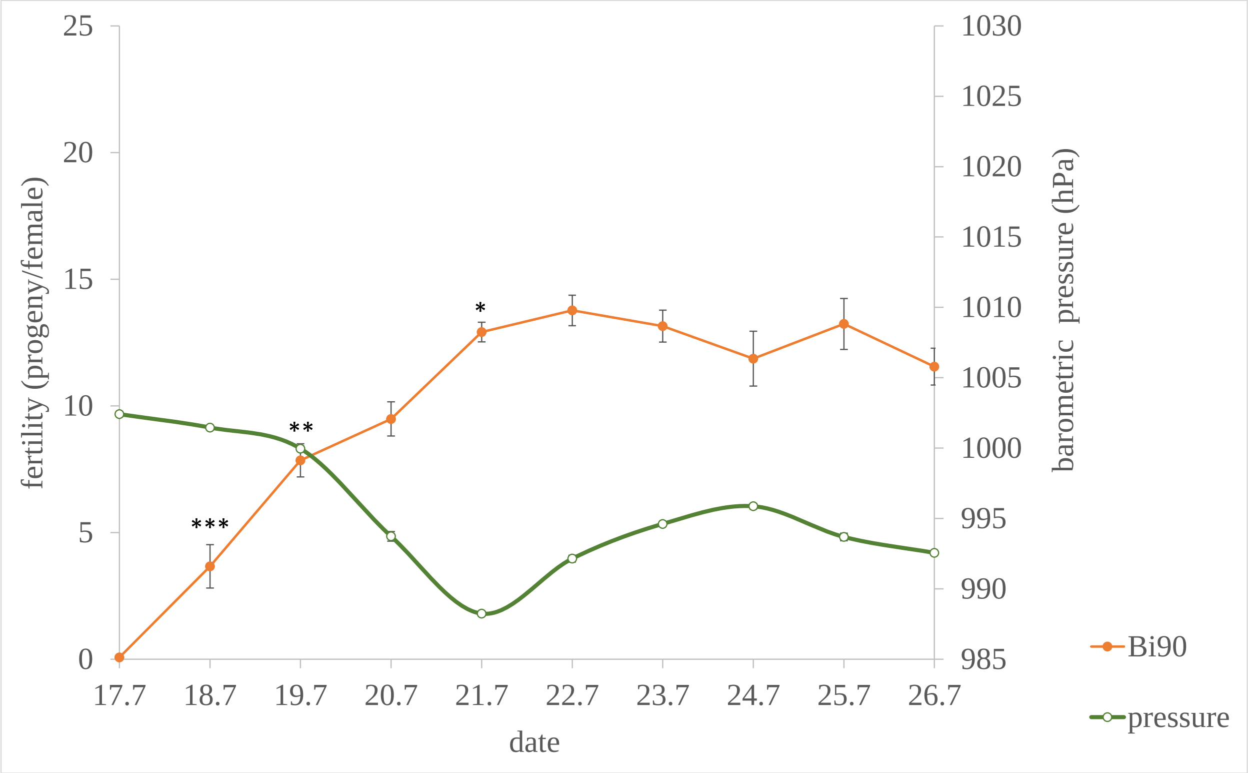

Figure S2. The fertility of *D. melanogaster* wild type strain Bi90 in comparison with variations of barometric pressure in Novosibirsk, Russia, in the end of July of 2020 (https://www.wunderground.com/history). Each point represents an average value of 10 tests (N=3 for each test) as means±s.e.m. The asterisks illustrate the post-hoc differences in comparison with the fertility level on previous day. One asterisk indicates $p<0.05$; two asterisks, $p<0.01$; three – $p<0.001$.
