# Supplementary material for: A Link between Atmospheric Pressure and Fertility of Drosophila Laboratory Strains
Source: Insects. 2021 Oct 18;12(10):947. doi: 10.3390/insects12100947 (PMC8538592; doi:10.3390/insects12100947)
Supplement: Supplementary file 1 [file insects-12-00947-s001.zip › Table S1.pdf]

Table S1. Principal component analysis (PCA) on meteorological data and fertility levels of Bi90 and 153 *D. melanogaster* strains.

	Principal component loadings					
**Variable**	**PC1**	**PC2**	**PC3**	**PC4**	**PC5**	**PC6**
**Temperature**	**+0.931**	+0.207	-0.184	-0.087	+0.121	+0.183
**Humidity**	**+0.703**	-0.026	+0.344	+0.579	-0.228	+0.007
**Wind speed**	**-0.757**	+0.329	+0.195	+0.423	+0.316	+0.050
**Air Pressure**	**-0.906**	-0.250	+0.145	-0.073	-0.257	+0.154
**Air Pressure change**	+0.287	**-0.726**	**+0.568**	-0.159	+0.206	+0.011
**Fertility (log)**	+0.078	**+0.725**	**+0.595**	-0.331	-0.068	-0.011
**Supplementary variable**	**PC1**	**PC2**	**PC3**	**PC4**	**PC5**	**PC6**
**Experiment***	**+0.939**	+0.105	-0.084	-0.037	-0.036	+0.219
**Within-day fertility variance***	+0.080	**+0.694**	**+0.539**	-0.280	-0.036	+0.017
	**PC1**	**PC2**	**PC3**	**PC4**	**PC5**	**PC6**
**PC Eigenvalue**	2.844	1.267	0.888	0.662	0.279	0.060
**Proportion of variance**	0.474	0.211	0.148	0.110	0.047	0.010
**Cumulative variance**	0.474	0.685	0.833	0.943	0.990	1.000

* - supplementary variables were not used in PCA to determine PCs
