# Supplementary material for: A Link between Atmospheric Pressure and Fertility of Drosophila Laboratory Strains
Source: Insects. 2021 Oct 18;12(10):947. doi: 10.3390/insects12100947 (PMC8538592; doi:10.3390/insects12100947)
Supplement: Supplementary file 1 [file insects-12-00947-s001.zip › Table S2.pdf]

Table S2. Principal component analysis (PCA) on meteorological data and fertility level of Bi90 *D. melanogaster* strain.

	Principal component loadings					
**Variable**	**PC1**	**PC2**	**PC3**	**PC4**	**PC5**	**PC6**
**Temperature**	**+0.926**	+0.204	-0.204	-0.043	+0.123	+0.207
**Humidity**	**+0.657**	-0.153	+0.459	+0.531	-0.227	+0.013
**Wind speed**	**-0.763**	+0.245	+0.316	+0.391	+0.318	+0.061
**Air Pressure**	**-0.911**	-0.235	+0.065	-0.109	-0.263	+0.172
**Air Pressure change**	+0.247	**-0.761**	+0.488	-0.289	+0.192	+0.016
**Fertility (log)**	+0.107	**+0.683**	+0.617	-0.369	-0.078	-0.004
	**PC1**	**PC2**	**PC3**	**PC4**	**PC5**	**PC6**
**PC Eigenvalue**	2.775	1.226	0.974	0.669	0.280	0.077
**Proportion of variance**	0.462	0.204	0.162	0.111	0.047	0.013
**Cumulative variance**	0.462	0.667	0.829	0.941	0.987	1.000

* - supplementary variables were not used in PCA to determine PCs
